# Determination of Oleanolic and Ursolic Acids in *Hedyotis diffusa* Using Hyphenated Ultrasound-Assisted Supercritical Carbon Dioxide Extraction and Chromatography

**DOI:** 10.1155/2015/450547

**Published:** 2015-05-18

**Authors:** Ming-Chi Wei, Yu-Chiao Yang, Show-Jen Hong

**Affiliations:** ^1^Department of Environmental Engineering & Science, Chia Nan University of Pharmacy and Science, Tainan 71710, Taiwan; ^2^Department and Graduate Institute of Pharmacology, Kaohsiung Medical University, Kaohsiung 80708, Taiwan

## Abstract

Oleanolic acid (OA) and ursolic acid (UA) were extracted from *Hedyotis diffusa* using a hyphenated procedure of ultrasound-assisted and supercritical carbon dioxide (HSC–CO_2_) extraction at different temperatures, pressures, cosolvent percentages, and SC–CO_2_ flow rates. The results indicated that these parameters significantly affected the extraction yield. The maximal yields of OA (0.917 mg/g of dry plant) and UA (3.540 mg/g of dry plant) were obtained at a dynamic extraction time of 110 min, a static extraction time of 15 min, 28.2 MPa, and 56°C with a 12.5% (v/v) cosolvent (ethanol/water = 82/18, v/v) and SC–CO_2_ flowing at 2.3 mL/min (STP). The extracted yields were then analyzed by high performance liquid chromatography (HPLC) to quantify the OA and UA. The present findings revealed that *H. diffusa* is a potential source of OA and UA. In addition, using the hyphenated procedure for extraction is a promising and alternative process for recovering OA and UA from *H. diffusa* at high concentrations.

## 1. Introduction

Historically, herbal medicines derived from plant extracts have been used to treat human diseases or maintain health. In recent years, plant research has received increasing amounts of attention worldwide; numerous studies describing the therapeutic properties of extracts from the plants used in traditional medicine have been developed, revealing the immense potential of medicinal plants.* Hedyotis diffusa* Willd, which belongs to the family Rubiaceae, is locally known as “Peh-Hue-Juwa-Chi-Cao” and is commonly known as Chinese tea [1, 2]. In traditional Chinese medicine, this plant is used extensively to treat hepatitis, tonsillitis, sore throat, appendicitis, urethral infection, and malignant tumors in the liver, lung, and stomach [3–7]. Recently, this herb has gained increasing amounts of attention regarding its usage as an antitumor herb in the liver, lungs, colon, brain, and pancreas [3, 4, 8, 9]. Ursolic acid (UA) and oleanolic acid (OA) are bioactive compounds that have been isolated from this herb and might be responsible for its therapeutic effectiveness. Both OA and UA have many important pharmacological activities including anticancer, chemopreventive, hepatoprotective, antiviral, antibacterial, antidiabetic, antioxidant, anti-inflammatory, and gastroprotective effects; these compounds display similar activities because their chemical structures are similar. Furthermore, OA and UA have a wide variety of antitumor activities, inhibiting hepatocellular carcinoma, prostate carcinoma, colorectal cancer, acute myelogenous leukemia, skin tumorigenesis, cervical carcinoma, and lung carcinoma [10]. These findings have made UA and OA attractive dietary supplements in the expanding health food market. Therefore, finding an effective and optimal method for isolating UA and OA from* H. diffusa* has become highly important.

Because plant extracts are composed of a complex mixture of phytochemical constituents that can cause interference within a sample and that can contain compounds similar to the bioactive analytes of interest, a strong matrix effect is often observed. Furthermore, the simultaneous quantification of OA and UA in plant extracts is difficult due to their structural similarities [11]. Therefore, selecting an effective chromatographic method for determining the target compounds is a key for the qualitative and quantitative analysis of the chemical constituents present in medical plants. Some studies have found that HPLC is the most convenient and comprehensive technique for separating triterpenic acids in plant extracts [12]. One goal of this work was to apply the HPLC method to determine the OA and UA contents of* H. diffusa*.

Traditionally, target compounds could be procured from herbs using organic solvent extraction along with maceration, heat-flux, and Soxhlet extraction techniques. However, a low selectivity or extraction yield, in addition to the long extraction times, toxic solvent residues, and degradation of temperature-sensitive compounds, may occur when using these techniques [12]. These issues are important for food, cosmetic, and medicinal extracts. Supercritical carbon dioxide (SC–CO_2_) extraction technology may be a viable alternative for solvent extraction methods. SC–CO_2_ is pushed beyond its critical point (7.38 MPa and 31.1°C) and has been recognized as an ideal extraction solvent. The most important advantages of this novel extraction method include the safe extraction of thermally labile compounds, shortened extraction times, the tunable selectivity, and solvent removal from the extracted materials [13, 14]. The extraction of OA and UA from various raw materials using SC–CO_2_ had been studied by Domingues et al. [15], Patinha et al. [16], and Yang et al. [10]. Furthermore, SC–CO_2_ was recently used as an attractive alternative extraction method for conventional liquid extraction in numerous areas including the food, pharmaceutical, and environmental engineering industries [17, 18].

Despite the above-mentioned advantages of SC–CO_2_ extraction, high-pressure equipment exhibits serious drawbacks when compared to traditional atmospheric pressure extraction techniques. Owing to its high pressure operating conditions, SC–CO_2_ extraction method is not only a high capital investment, but also a mechanical stirring difficult to be applied, resulting in a decrease in extraction kinetics. This limitation is often overcome by proper pretreatment of the sample, such as sonication to the entrainer prior to the extraction. Therefore, the use of combinatory and hyphenated SC–CO_2_ with other techniques can achieve good extraction efficiency with required selectivity in the same or shorter duration using milder conditions than that of solely SC–CO_2_ extraction. Recently, several studies investigating the combination application of SC–CO_2_ extraction and ultrasound-assisted extraction of target compounds from raw matrices have been published [14]. In this study, a hyphenated process (HSC–CO_2_) consisting of ultrasound-assisted static stage, followed by SC–CO_2_ dynamic extraction (without ultrasound), was used for the simultaneous separation of OA and UA from* H. diffusa*. In addition, the effects of the HSC–CO_2_ extraction parameters such as the pressure, temperature, cosolvent percentage, SC–CO_2_ flow rate, and dynamic extraction time were evaluated to obtain the highest extraction yield, and the precision of the method was examined. The analyses of the extracts were performed using high performance liquid chromatography (HPLC) with ultraviolet/visible (UV/vis) multiwavelength detection. The results were compared with those obtained using conventional extraction techniques. The aim of this study was to develop a simpler and more environmentally friendly technique with high efficiency, low toxicity, and high selectivity for natural products.

## 2. Materials and Methods

### 2.1. Plant Materials

The dried whole-plant materials from* H. diffusa* (samples HD1 to HD3) were kindly provided by Chuang Song Zong Pharmaceutical Co. Ltd. (Kaohsiung, Taiwan). The air-dried whole plants were pulverized in a knife mill, and the plant particles were sieved to produce samples with sizes of 0.925, 0.725, 0.550, 0.355, and 0.210 mm (mean diameter). These fractions were subsequently packed in plastic bags and stored at 4°C for later use. The moisture contents (% of dry weight basis) were determined by drying at 105°C to a constant mass and were 11.32%, 10.41%, and 11.25%. All of the yields and compositions were calculated based on moisture-free conditions and represent the mean values of at least six experiments.

### 2.2. Chemicals and Reagents

Both OA and UA were purchased as HPLC reference standards from Sigma Chemical Co. (St. Louis, MO, USA). The methanol, ethanol, acetone, acetonitrile, ethyl acetate, *n*-hexane, and 85% phosphoric acid were purchased from Merck Co. (Darmstadt, Germany). Carbon dioxide was purchased from Yun-Shan Gas Co. Ltd. (Tainan, Taiwan) and was used during the SC–CO_2_ extraction.

### 2.3. Hyphenated Procedure of Ultrasound-Assisted and Supercritical Carbon Dioxide (HSC–CO_2_) Extraction

**Figure 1:**
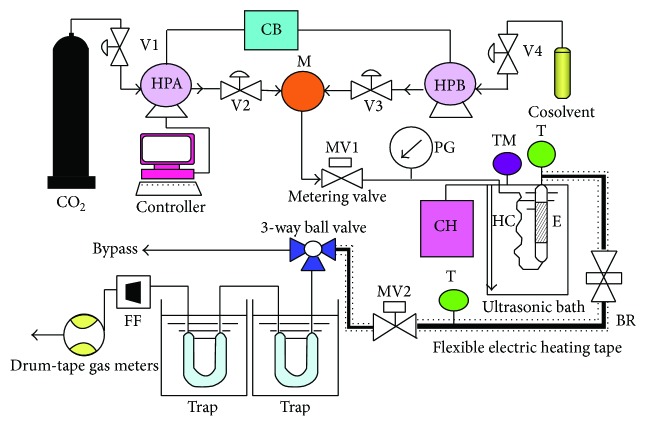
Schematic diagram of the HSC–CO_2_ extraction apparatus. V1, V2, V3, and V4: stopping valve (on-off valve); HPA, HPB: syringe pump; M: mixer; CB: circulation bath; CH: circulating heater; MV1, MV2: micrometering valve; HC: heating coil; E: extraction vessel; PG: pressure gauge; BR: backpressure regulator; FF: float flowmeter T: thermocouple; and TM: mercury-in-glass thermometer.

As shown in Figure 1, the HSC–CO_2_ extraction apparatus was a semicontinuous flow, high-pressure system. The major parts of the apparatus were a CO_2_ cylinder, two syringe pumps (ISCO 260D; 100DX, Lincoln, NE, USA), and a controller (ISCO SFX 200, Lincoln, NE, USA). The herb sample (10.0 g) was thoroughly mixed with 2 mm stainless steel balls before being placed into the 43 mL extraction vessel. The extraction vessel was then immersed in an ultrasonic bath with a working frequency of 40 kHz and 185 W of power (Branson B-33810E-DTH, USA), which was controlled by an electrical heater (Thermo Haake, model DC10, USA) to within ±0.1°C, and the system was brought to the desired temperature. The extraction system was operated with a static period of 15 min (with ultrasound-assisted) under working conditions to allow contact between the samples and the supercritical solvent, which was followed by dynamic extraction for 20–160 min (without ultrasound-assisted). More details regarding the equipment and its operation can be found in a previous work [13]. To ensure the accuracy of the experimental data, this extraction process was repeated six times for each sample. The data are presented as the means ± standard deviation (SD).

### 2.4. Heat-Reflux Extraction

Heat-reflux extraction (HRE) was also investigated and compared to the HSC–CO_2_ extraction process and has already been described in detail [19–21].

### 2.5. HPLC Analysis

A double-beam U-300 UV/vis spectrophotometer (Hitachi, Japan) was originally used to determine the maximum absorbance wavelength for each analyte [12]. The HPLC analysis of OA and UA was carried out on a Jasco HPLC system (Jasco, Tokyo, Japan) with a LiChrospher C-18 analytical column (250 mm × 4 mm i.d., 5 *μ*m particle size; Merck, Darmstadt, Germany). The mobile phase was composed of acetonitrile (solvent A) and water containing 0.1% phosphoric acid (solvent B) and was used under the following gradient conditions: 0–25 min at 22-23% (solvent A) and 1.0–1.5 mL/min, 25–40 min at 23% (solvent A) and 1.5–1.0 mL/min, and 40–60 min at 23–90% (solvent A) and 1.0 mL/min. The column was maintained at 40°C and the effluent was monitored at 210 nm with an intelligent UV/vis multiwavelength detector (Jasco MD-910, Jasco, Tokyo, Japan). The peaks for the target compounds within the extracts were identified based on the retention time and chromatographic behavior versus the authentic standards. The quantity of the target compounds was calculated by comparing their peak area to that of the standards.

### 2.6. Statistical Analysis

All of the yields and composition analyses were calculated on a moisture-free basis. The mean value and SD were calculated based on six experiments. The results are expressed as the means ± SD. An analysis of variance (ANOVA) was carried out using Tukey's method with a significance level of *P* < 0.05 using Microsoft Office Excel 2010 (Microsoft CO., USA) and Origin software version 6.1 (Origin Lab CO., Northampton, MA, USA).

## 3. Results and Discussion

### 3.1. Qualitative and Quantification Analysis of the Extracts by HPLC

**Figure 2:**
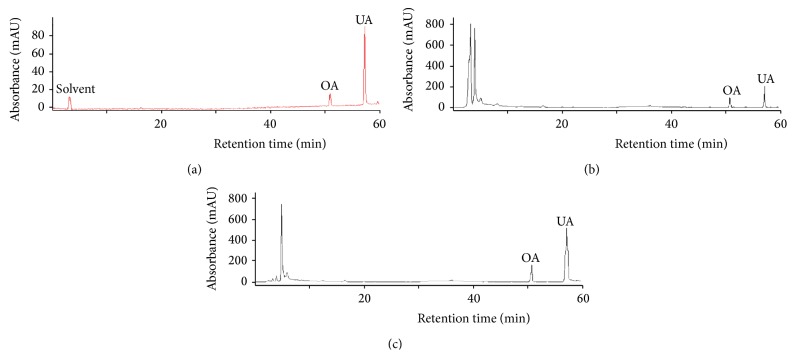
HPLC chromatograms of the standard solution (a), an extract obtained using HRE (b), and an extract obtained using HSC–CO_2_ extraction (c).

The HPLC profiles are reported in Figure 2, which illustrates the separation of OA and UA.  Figure 2(a) shows that the standard substances have retention times of 50.44 ± 0.06 min and 56.62 ± 0.08 min for OA and UA, respectively. The HPLC chromatograms of the* H. diffusa* extracts using HRE and HSC–CO_2_ with aqueous ethanol as the cosolvent are also shown in Figures 2(b) and 2(c), respectively. Many other peaks appear in the HPLC chromatograms of the* H. diffusa* extracts. However, Figures 2(b) and 2(c) revealed that no interference peaks from the endogenous constituents of the HRE and HSC–CO_2_ extracts were found in the region containing the investigated compounds; therefore, good separation could be obtained, and the OA and UA were assigned the retention times of 50.44 ± 0.06 min and 56.62 ± 0.08 min, respectively. The chromatographic peaks of OA and UA were confirmed by comparing their retention time and their spectral characteristics against those of the authentic standards. Furthermore, the contents of the analyzed compounds from the crude extracts of* H. diffusa* were quantitated based on the calibration curves for the standard compounds.

### 3.2. Heat-Flux Extraction

The type of solvent affects the extraction efficiency. First, to determine the effect of the solvent type on the yield of the studied compounds, two nonpolar solvents (chloroform and *n*-hexane) and two polar solvents (ethanol and water) under HRE were tested. The other parameters including the 60 min extraction time, temperature of 75°C, solvent-to-raw material ratio of 16 mL/g, mean particle size of 0.355 mm, and stirring rate of 300 rpm remained constant throughout the study. The solvents differ in polarity; therefore, they should alter the extraction performance. After comparing the tested extraction solvents, the highest amounts of target compounds were obtained when chloroform and ethanol were used; the two triterpenic acids are insoluble in water and in hexane (Table 1). The polarity of chloroform and ethanol relative to the discussed compounds explains these results. Additionally, due to the medium polarity OA and UA, the nonpolar hexane and polar water were not efficient for extraction. However, chloroform is a toxic solvent; therefore, it is not suitable for use in the food, pharmaceutical, and cosmetic industries. However, the most commonly used extraction agent is ethanol due to its low toxicity, making it acceptable for practical use in the food, cosmetic, and pharmaceutical industries. Additionally, ethanol can be mixed with water in different ratios, and it was consequently chosen as the extraction solvent for the OA and UA from* H. diffusa*.

The influence of the aqueous ethanol concentration on the yields of OA and UA is shown in Table 1. The data indicate that 70% and 90% ethanol in water were the best solvent composition for extracting OA and UA selectively, respectively. Therefore, ethanol diluted with water increased the yield versus pure ethanol or water because the lower water content increased the swelling effect of the plant tissue matrix, decreased the viscosity of solvent, and improved the mass transport from the material, facilitating the extraction of the OA and UA. However, utilizing more than 50% water in aqueous ethanol increased the polarity of the mixed solvent beyond the point at which it was suitable for extracting OA and UA; therefore, the yield decreased. Similar outcomes were observed during the UAE of OA and UA from* Scutellaria barbata* D. Don [20], schisandrin B from* Schisandra chinensis* (Turcz.) Baill seeds [22], and cepharanthine from* Stephania rotunda* Lour [23].

### 3.3. Extraction with Carbon Dioxide

One of our objectives was to compare the HRE and SC–CO_2_ extractions while using both nonpolar and polar solvents. During our study, pure SC–CO_2_ was initially used to investigate the extraction of OA and UA from* H. diffusa*. To achieve the highest extraction efficiencies, several factors were investigated such as the extraction pressure (10.4–30.0 MPa), extraction temperature (40–70°C), static extraction time (10–30 min), dynamic extraction time (10–180 min), mean particle size (0.925–0.210 mm), and SC–CO_2_ flow rate (0.6–2.5 mL/min (STP)). However, SC–CO_2_ without a cosolvent was not selective for OA and UA, even under different extraction pressures and temperatures. OA and UA were not detected under any conditions analyzed at different extraction pressures (10.4–30.0 MPa) and temperatures (40–70°C). The extraction using pure SC–CO_2_ generated yields similar to those obtained when *n*-hexane was used as a solvent during HRE (Table 1).

### 3.4. Extraction with Carbon Dioxide: Aqueous Ethanol Mixtures

As mentioned above, OA and UA are polar compounds, rendering extractions using only SC–CO_2_ ineffectively (Table 1). Consequently, a polar cosolvent should be used to enhance the selectivity and to increase the extraction efficiency for the selected components. Based on the preliminary experiments, adding a small amount of aqueous ethanol to the SC–CO_2_ can significantly enhance the extraction efficiency and, consequently, increase the extraction yield. This method was used to improve the yields of OA and UA from* S. barbata* D. Don [10]. During this study, the mean particle size, water content in the ethanol cosolvent, percent ratio of the cosolvent (aqueous ethanol) in the mixed fluid, and the extraction conditions including the SC–CO_2_ flow rate, extraction time, temperature, and pressure are the significant variables when extracting OA and UA from* H. diffusa*.

#### 3.4.1. Effect of the Cosolvent Contents

**Figure 3:**
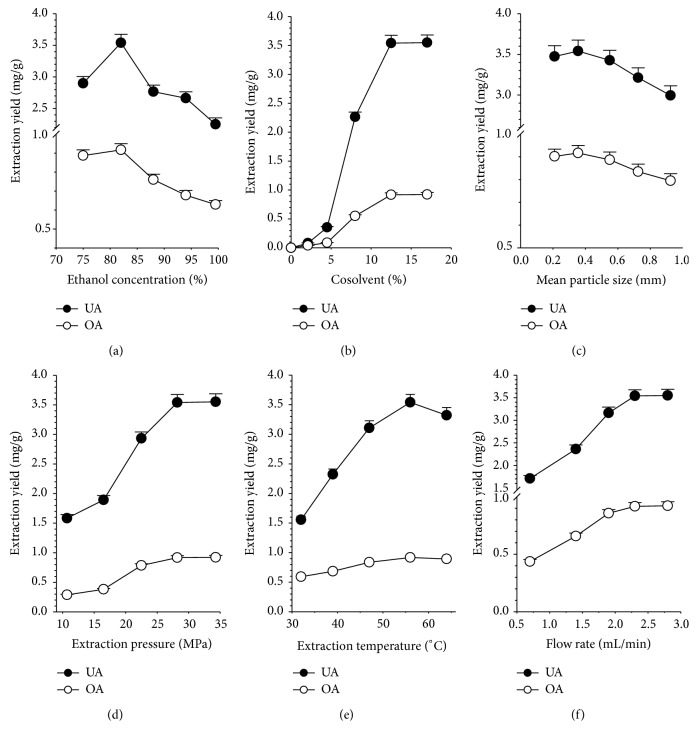
Effects of cosolvent contents (a), co-solvent percentage (b), mean particle size (c), extraction pressure (d), extraction temperature (e), and SC–CO_2_ flow rate (f) on the extraction yields of OA and UA from* H. diffusa* using HSC–CO_2_ extraction.

The effect of the water content of the ethanol cosolvent (water/ethanol = 0, 6/94, 12/88, 18/82, and 25/75, v/v) on the extraction yield was examined at a mean particle size of 0.355 mm, a temperature of 56°C, a pressure of 28.2 MPa, a static time of 15 min (with ultrasound-assisted), a dynamic time of 110 min, and a CO_2_ flow rate of 2.3 mL/min while using 12.5% cosolvent (aqueous ethanol); the results are shown in Figure 3(a). Adding an aqueous ethanol cosolvent can greatly improve the extraction efficiency due to the enhanced solubility of OA and UA after increasing the polarity of the SC–CO_2_. Moreover, aqueous ethanol cosolvent accelerates the desorption process by reducing the interactions between the solutes and the sample matrix, competing with the solutes for active binding site and disrupting the matrix structure. As observed in Figure 3(a), the yield of OA and UA increased significantly when the water (v/v) content of the aqueous ethanol increased from 0 to 18% (v/v). After 110 min, a slight decrease was observed in the yield of the OA and UA when the water (v/v) content of the aqueous ethanol was 25% throughout the extraction period because the higher water content increases the polarity of the cosolvent, facilitating the extraction of OA and UA. Consequently, when the polarity of cosolvent is too high (25%), the capability for extracting the studied components will decrease. Similar results were also observed during SC–CO_2_ extraction of OA and UA from* S. barbata* D. Don [10] and of flavonoids from hops (*Humulus lupulus *L.) [24]. Therefore, the best cosolvent composition for OA and UA extraction from* H. diffusa* was 82% ethanol.

#### 3.4.2. Effect of Modifier (82% Aqueous Ethanol) Percentage

The major limitation of utilizing SC–CO_2_ is its inability to dissolve polar analytes, even at very high densities. The most effective way to eliminate this limitation is to add a polar solvent to the SC–CO_2_, increasing its polarity while decreasing the strength of the interaction between the analyte and the matrix; this method also efficiently displaces the polar analytes from the matrix. However, the percent ratio of the modifier in a mixed fluid (SC–CO_2_ + modifier) is an important parameter. To test the effects of the modifier percentages on the extraction efficiency, the modifier (82% ethanol) content was explored while maintaining the following conditions: the mean particle size, dynamic time, CO_2_ flow rate, pressure, and temperature of 0.355 mm, 110 min, 2.3 mL/min, 28.2 MPa, and 56°C, respectively. Various percent ratios of the modifier (82% ethanol) in a mixed fluid (4.5, 8.0, 12.5, and 17% (v/v)) were utilized during the HSC–CO_2_ extraction of OA and UA from* H. diffusa*. Different extraction results could be expected when manipulating the ratios of the liquid cosolvent, and the effects of the cosolvent percentage on the extraction efficiency of OA and UA using HSC–CO_2_ are shown in Figure 3(b). The amount of OA and UA extracted per gram of* H. diffusa* increases when increasing the cosolvent content to certain level (12.5%) before leveling off at higher percentages (17%). Similarly, Içen and Gürü reported that the yields significantly increased when increasing the ratio of alcohol/CO_2_ to 5.2% but did not vary when increasing the ratio further during an extraction using ethanol with supercritical carbon dioxide for caffeine from tea stalk and fiber waste [25]. The various percentages of the cosolvent exhibited different effects when changing the polarity of the SC–CO_2_; therefore, diverse effects were observed when enhancing the solubility of the polar analytes. The best extraction yield is obtained when the polarity of the mixed fluid (SC–CO_2_ + cosolvent) and polar analytes are coincident. In this study, the results indicated that the best cosolvent (82% ethanol, v/v) percentage for extracting OA and UA was 12.5% (v/v). Moreover, adding 12.5% aqueous ethanol (82% ethanol, v/v) as the cosolvent increases the yields of OA and UA, indicating that the cosolvent percentage could affect the destruction of the cellular walls and improve the mass transfer from inside the cells. Therefore, 12.5% aqueous ethanol (82% ethanol, v/v) was the best cosolvent system because it produced higher yields of OA and UA.

#### 3.4.3. Effect of the Mean Particle Size

The HSC–CO_2_ extraction yields of OA and UA from* H. diffusa* samples with different particle sizes (0.925, 0.725, 0.550, 0.355, and ≤0.210 mm) at 56°C, 28.2 MPa, a static time of 15 min (with ultrasound-assisted), a dynamic time of 110 min, a CO_2_ flow rate of 2.3 mL/min, and a cosolvent (82% ethanol, v/v) percentage of 12.5% are shown in Figure 3(c). As expected, the yields of the OA and UA increased significantly when the particle size of* H. diffusa* decreased from 0.925 to 0.355 mm. This increase occurred because the smaller particle size shortens the diffusion paths in solid matrix decreasing the intraparticle resistance toward diffusion while exposing more cells to the supercritical solvent; therefore, these extraction yields for the OA and UA were higher than those for the larger sample diameters. However, a slight decrease in the yields of the OA and UA was observed for the 0.210 mm particles throughout the extraction period because the small particles aggregated during extraction, causing the fluid to channel or short circuit. Moreover, the small particle size was implicated in the readsorption of extracted solutes onto the matrix surface, which may also be responsible for the effect of the particle sizes on the yield of OA and UA. Similar variations were also obtained during the UAE of antioxidants from pomegranate marc [26] and during the ultrasound-assisted SC–CO_2_ extraction of oils from* Syzygium aromaticum* flower buds (clove) [14]. Therefore, the best mean particle size was 0.355 mm, which was adopted for the subsequent experiments.

#### 3.4.4. Effect of the Extraction Pressure

The effects of the extraction pressure on the yields of OA and UA when the mean particle size was 0.355 mm, the temperature was 56°C, the static time was 15 min (with ultrasound-assisted), the dynamic time was 110 min, the CO_2_ flow rate was 2.3 mL/min and the cosolvent (82% ethanol, v/v) percentage was 12.5% are shown in Figure 3(d). Notably, the yields of OA and UA increased when increasing the pressure from 10.7 to 28.2 MPa due to the increased density of SC–CO_2_; modulating the density of the SC–CO_2_ alters the solubility of the solute, enhancing the extraction efficiency. However, the yields of OA and UA plateaued when the pressure was raised from 28.2 to 34.3 MPa. The pressure can change the density, viscosity, and diffusion characteristics of the SC–CO_2_. Theoretically, the higher the pressure, the larger the density of SC–CO_2_; however the diffusivity of the fluid may decrease, lowering the extraction yield because the interaction between SC–CO_2_ and solid matrix is decreased. Moreover, increasing the extraction pressure packs the solid matrix more tightly while decreasing the void fraction, further reducing the penetration of SC–CO_2_ into the solid matrix and decreasing the extraction efficiency. Therefore, this study reveals the predominant effect of pressure on the amount of OA and UA extracted by SC–CO_2_; for pressures ranging from 10.7 to 28.2 MPa, increasing the density of SC–CO_2_ is presumably the primary mechanism for increasing the yields of the triterpenic acids. However, the decreased effective diffusivity and mass transfer coefficient should affect the yields of OA and UA at 34.3 MPa more than the increased density. Similar results were reported by Içen and Gürü; their caffeine extraction yield increased considerably with increasing pressure up to 250 bar but did not vary significantly at higher pressures [25]. Similar findings were also obtained during the extraction of nobiletin and tangeretin from* Citrus depressa* Hayata [27]. However, using a higher pressure is not cost-effective due to the high operating costs and energy consumption. Therefore, a pressure of 28.2 MPa is appropriate for increasing the efficiency of the OA and UA extraction from* H. diffusa* in SC–CO_2_.

#### 3.4.5. Effect of the Extraction Temperature

To examine the effect of varying the temperature (32, 39, 47, 56, and 64°C), HSC–CO_2_ extraction experiments were carried out at a pressure of 28.2 MPa, a static time of 15 min (with ultrasound-assisted), a dynamic time of 110 min, a CO_2_ flow rate of 2.3 mL/min, and a cosolvent (82% ethanol, v/v) percentage of 12.5% while using sample HD3 with a mean particle size of 0.355 mm and a moisture content of 11.25%. As shown in Figure 3(e), when the temperature increases from 32 to 56°C, the yield of OA and UA increases from 0.592 to 0.917 mg/g dry plant and from 1.555 to 3.540 mg/g dry plant, respectively; if the extraction temperature is increased to 64°C, the yields decrease. Increasing the temperature at the given pressure decreases the density of the SC–CO_2_ density, while the volatile properties of the analytes and the desorption of the substances from the matrix increase. Therefore, this study speculated that, from 32 to 56°C, the influence of the temperature on the yield is predominated by the solid vapor pressure and desorption effects more than the variations in the SC–CO_2_ density; at temperatures above 56°C, the effects of the density predominate. These results agree with the SC–CO_2_ extraction of Amaranth seed oil by Westerman et al. [28] and of andrographolide from* Andrographis paniculata* by Kumoro and Hasan [29]. Therefore, a lower extraction temperature (56°C) is recommendable to maximize the economy and yield of the process.

#### 3.4.6. Effect of the SC–CO_2_ Flow Rate

Another major parameter affecting the efficiency and overall economy of HSC–CO_2_ extraction is the CO_2_ flow rate. If the extractions in the experiments were performed at a constant temperature and pressure, a low CO_2_ flow rate resulted in a longer residence time and vice versa. A critical analysis of the literature reveals that a longer residence time allows the SC–CO_2_ to remain in the extraction vessel longer, allowing it to remain in contact with and diffuse through the pores of the raw materials while increasing the extraction yields [30]. SC–CO_2_ flow rates too low to generate a sufficient amount of SC–CO_2_ to extract the target compounds lower the extraction yields [31]. Increasing the CO_2_ flow rate not only lowered the residence time but also increased the number of SC–CO_2_ molecules in contact with the solute, increasing intermolecular interactions between the SC–CO_2_ and the solute and enhancing the dissolution of the solute. However, when the SC–CO_2_ flow rate increased, it flows through the raw materials at high velocities instead of diffusing through the sample matrix, flowing around the raw materials through channels and consequently limiting the contact necessary for extracting the target compounds [30, 32]. Therefore, characterizing the kinetics and optimal CO_2_ flow rates of the extraction process is critical to attain a complete extraction while accounting for the efficiency and cost of the extraction.

To minimize the extraction time and the related costs of the HSC–CO_2_ extraction procedure, four flow rates (0.7, 1.4, 1.9, 2.3, and 2.8 mL/min (STP)) were carried out while assessing the yields of the OA and UA; these experiments were the same as those previously mentioned, except that the CO_2_ flow rate was varied.  Figure 3(f) reveals that when the flow rate was increased from 0.7 to 2.3 mL/min, the extraction rate of OA and UA increased but did not vary significantly above that range (2.8 mL/min). Similar phenomena were also reported for the extraction of nimbin from neem seeds using SC–CO_2_ [33]. Therefore, 2.3 mL/min CO_2_ value was selected as the flow rate for the following experiments.

#### 3.4.7. Effect of the Extraction Time

**Figure 4:**
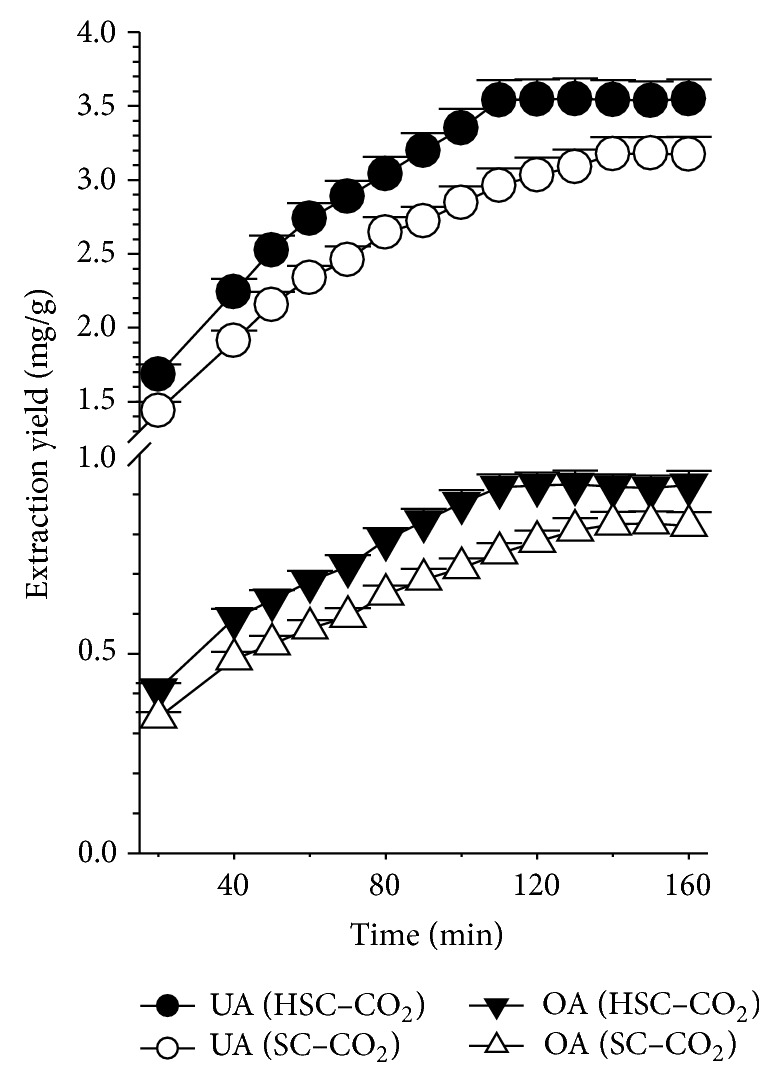
Effect of dynamic extraction time on the extraction yields of OA and UA from* H. diffusa* using HSC–CO_2_ and SC–CO_2_ extractions. Conditions: 0.355 mm, 56°C, 28.2 MPa, 2.3 mL/min, and 12.5% cosolvent (82% aqueous ethanol).

To achieve high extraction efficiencies, a primary extraction step in static mode (with ultrasound-assisted for 15 min and without ultrasound-assisted for 25 min) was performed, allowing the SC–CO_2_ to penetrate the matrix more thoroughly than in dynamic mode. This step was followed by a dynamic extraction to enhance the solubility of OA and UA in the SC–CO_2_.  Figure 4 shows the effects of the dynamic extraction time on the yields of OA and UA when the mean particle size was 0.355 mm, the temperature was 56°C, the pressure was 28.2 MPa, and the CO_2_ flow rate was 2.3 mL/min while 12.5% cosolvent (82% ethanol, v/v) was used. The experimental results revealed that the dynamic time strongly affected the HSC–CO_2_ extraction yields for OA and UA.  Figure 4 shows that when the dynamic extraction time is increased from 20 to 110 min, the balance of the extraction shifted to favor the extraction of OA and UA. However, further increases in the dynamic extraction time (120–150 min) did not affect the yield of target compounds significantly; therefore, 110 min was selected for the extraction time. Özkal et al. [34] observed similar behavior during the supercritical extraction of hazelnut oil at 30–60 MPa and 40–60°C with 2 mL/min SC–CO_2_.

The experimental conditions of conventional SC–CO_2_ extraction were the same as those aforementioned HSC–CO_2_ extraction except that the static extraction time of 25 min was without ultrasound-assisted. According to the result obtained (Figure 4), extraction yield was enhanced by increasing the dynamic extraction time (20–140 min). However, since the difference between the extraction yields obtained for 140 and 160 min was not significantly different, 140 min is a reasonable time to use for the conventional SC–CO_2_ extraction. The results shown in Figure 4 indicate that HSC–CO_2_ extraction time of 110 min was sufficient to obtain the maximum yield, while 140 min was required for conventional SC–CO_2_ extraction to reach the maximum yield. Furthermore, HSC–CO_2_ extraction significantly improved the extraction yield, compared to conventional SC–CO_2_ extraction. Since swelling and hydration could be accelerated by ultrasonic in static stage, this results in a probable enlargement in the pores of the cell walls, leading to a better mass transfer of intracellular products into SC–CO_2_. In addition, the rupture of cell walls by microjet may also cause an increased penetration rate of SC–CO_2_ into tissue. Therefore, HSC–CO_2_ extraction technique allowed the discussed compounds to dissolve in SC–CO_2_ at a higher rate, thereby boosting yield in a relevant shorter time.

### 3.5. Comparison of the Different Extraction Methods

To evaluate the extraction efficiency of the HSC–CO_2_, a HRE was also used to extract the OA and UA from* H. diffusa*. The extraction yields for the individual techniques are compared in Table 2. The two triterpenic acids are insoluble in water and nonpolar solvents such as hexane and pure SC–CO_2_ but are freely soluble in alcoholic solvents. Furthermore, the HSC–CO_2_ and HRE results revealed that the extraction conditions for OA were close to those of UA. This result is supported by the similarity of the chemical structures of OA and UA. The best extraction conditions for HRE were as follows: 70% ethanol for OA or 90% ethanol for UA, a 1 : 16 ratio of material to liquid, a particle size of 0.355 mm, and an extraction time of 60 min at 75°C. Under the best HRE conditions, the yields of OA and UA were 0.762 ± 0.030 and 2.964 ± 0.094 mg/g (4 extraction cycles), respectively. Concurrently, the highest OA (0.917 mg/g dry plant) and UA yields (3.540 mg/g dry plant) of HSC–CO_2_ extraction were obtained at 56°C, 28.2 MPa, 2.3 mL/min CO_2_, a particle size of 0.355 mm, and a cosolvent (ethanol/water = 82/18, v/v) percentage of 12.5%. The yields of OA and UA from the HSC–CO_2_ extraction were significantly higher than the yields from HRE. Compared to conventional HR, HSC–CO_2_ extractions can also shorten the extraction time (125 versus 240 min), lower the extraction temperature (56°C versus 75°C), and decrease the amount of solvent consumed. These improvements might be very interesting for industrial processes because HSC–CO_2_ extraction would improve both process rates and yields, consequently reducing the processing times and costs. Additionally, the HPLC results (Figure 2) revealed that the extracts obtained using HSC–CO_2_ contained lower levels of impurities compared to the extracts obtained through the classical extraction procedure; therefore, SC–CO_2_ extraction is more selective for OA and UA, generating better quality extracts. This improvement may be attributed to the fact that the lower HSC–CO_2_ extraction temperature decreases the impurity contents while increasing the overall purity. This proposed behavior was supported by the increased extractability of OA and UA from the plants when using the HSC–CO_2_ extraction process versus the classical extraction. Therefore, using HSC–CO_2_ with aqueous ethanol as the cosolvent to extract OA and UA from* H. diffusa* was preferable and was considered the most environmentally friendly extraction method.

## 4. Conclusions

A hyphened ultrasound-assisted SC–CO_2_ was used to extract OA and UA from* H. diffusa*. The extraction conditions were evaluated, and the yields of OA and UA reached 0.917 and 3.540 mg/g of dry material, respectively. The HSC–CO_2_ extraction generated higher yields of OA and UA than the other conventional extraction methods while saving time and organic solvent. Furthermore, an HPLC analysis revealed that the purity of the OA and UA obtained from the current HSC–CO_2_ extraction procedure was higher than that obtained from a conventional solid-liquid extraction. Therefore, food grades OA and UA can be obtained in high yields at a low cost. Therefore, HSC–CO_2_ extraction with aqueous ethanol as a cosolvent might be a viable new approach for obtaining valuable components, such as OA and UA, from* H. diffusa*; this process is also considered the most environmentally friendly extraction method.

## Figures and Tables

**Table 1:** Yields of the investigated components obtained through the heat-reflux extraction (HRE) method with various solvents (one extraction cycle) and the pure SC–CO_2_ extraction method.

Methods/solvents	Extraction yield (mg/g)^a^	
	OA	UA
HRE^b^		
Nonpolar		
Chloroform	0.582 ± 0.023	2.580 ± 0.105
*n*-Hexane	Not detected	Not detected
polar		
0% Ethanol (water)	Not detected	Not detected
10% Ethanol	Not detected	Not detected
20% Ethanol	Not detected	Not detected
30% Ethanol	Not detected	Not detected
40% Ethanol	Not detected	Not detected
50% Ethanol	Not detected	Not detected
60% Ethanol	0.544 ± 0.021	1.171 ± 0.045
70% Ethanol	0.661 ± 0.025	1.674 ± 0.060
80% Ethanol	0.650 ± 0.024	2.283 ± 0.082
90% Ethanol	0.629 ± 0.022	2.601 ± 0.093
95% Ethanol	0.623 ± 0.025	2.340 ± 0.092
99.5% Ethanol	0.599 ± 0.024	2.211 ± 0.081
Pure SC−CO_2_^c^	Not detected	Not detected

^a^Values are mean ± SD of six replications and are calculated on plant dry weight basis (HD3).

^b^The experimental conditions are described in the experimental section.

^c^Pure SC−CO_2_ conditions: extraction pressure at 10.4–30.0 MPa, extraction temperature at 40–70°C, a static extraction time of 30 min, a dynamic extraction time of 10–180 min, mean particle size at 0.096–0.925 mm, and CO_2 _flow rate at 0.6–2.5 mL/min (STP).

**Table 2:** Comparison of extraction yields and extraction conditions obtained by the HRE, SC–CO_2_ and HSC–CO_2_ methods.

Extraction parameters	Extraction mode		
	HRE	SC–CO_2_	HSC–CO_2_
Herbal sample	HD3	HD3	HD3
Mean particle size (mm)	0.355	0.355	0.355
Plant weight (g)	5	5	10
Stirring rate (rpm)	300	—	—
Static extraction time (min)	—	25	15
Ultrasonic frequency (kHz)	—	—	40
Duty cycle (%)	—	—	79
Dynamic time (min)	—	140	110
Extraction time (min)	60 × 4 (4 cycles)	165	125
Extraction temperature (°C)	75 (Boiling point)	56	56
Extraction pressure (MPa)	—	28.2	28.2
Liquid/solid ratio (mL/g)	16	64.4	50.6
CO_2_ flow rate (mL/min)	—	2.3	2.3
Extraction cycles	4	—	—
Cosolvent (v/v%)	—	82% ethanol	82% ethanol
Percentage of cosolvent (82% ethanol) in SC–CO_2_	—	12.5%	12.5%
OA:			
Yield (mg/g)^a^	0.762 ± 0.030	0.824 ± 0.032	0.917 ± 0.033
Ethanol (v/v%)^b^	70%	—	—
RSD (%)^c^	3.94	3.88	3.696
UA:			
Yield (mg/g)^a^	2.964 ± 0.094	3.175 ± 0.114	3.540 ± 0.135
Ethanol (v/v%)^b^	90%	—	—
RSD (%)^c^	3.17	3.59	3.825

^a^Values are written as the mean ± SD of six replications and are calculated based on plant dry weight basis.

^b^Ethanol concentration in water (v/v%).

^c^RSD (%) = (SD/mean) × 100.
